# *Holarrhena pubescens* (Kutaj) bark extract mitigates SDS-induced IBD-like pathologies in *Drosophila melanogaster* by preserving barrier integrity and antioxidant defense

**DOI:** 10.3389/fphar.2026.1841227

**Published:** 2026-06-24

**Authors:** Acharya Balkrishna, Simran Kauts, Sandeep Kumar, Gyanendra Singh Sengar, Sudeep Verma, Rishabh Dev, Anurag Varshney

**Affiliations:** 1 Drug Discovery and Development Division, Patanjali Research Foundation, Haridwar, India; 2 Department of Allied and Applied Sciences, University of Patanjali, Haridwar, India; 3 Patanjali Yog Peeth (UK) Trust, Glasgow, United Kingdom

**Keywords:** *Drosophila melanogaster*, gut integrity, *Holarrhena pubescens*, IBD, smurf assay

## Abstract

Inflammatory bowel disease (IBD) is a group of chronic conditions characterized by relapsing inflammation of gastrointestinal tract. Conventional treatments for IBD are confronted with various challenges including treatment failure, a therapeutic ceiling that restricts effectiveness and associated adverse effects. Present study employed *Drosophila melanogaster* as a model organism to investigate the protective effect of *Holarrhena pubescens* Wall. ex G. Don (Kutaj) Bark Extract (HpBE) against sodium dodecyl sulfate (SDS)-induced IBD-like etiologies. Ultra-high performance liquid chromatography (UHPLC) identified Epigallocatechin (EGC), Epicatechin (EC) and Protocatechuic acid as key phytometabolites in HpBE. On LCMS/MS-TQ analysis, these phytometabolites were also detected in whole body lysates of *Drosophila* treated with HpBE, confirming their absorption. HpBE was tolerated well in *Drosophila*, enhanced survival and mitigated SDS-induced intestinal injury, as demonstrated by smurf and trypan blue dye exclusion assays. Importantly, treatment with HpBE improved feed uptake, restored excretory function and prevented weight loss. Notably, HpBE also prevented the general sickness associated with inflamed gut, as evident from enhanced climbing activity. SDS-exposed flies that received HpBE, exhibited enhanced antioxidant defence and preserved cytoskeletal integrity of gut, as evidenced by increased GSH levels, SOD activity and phalloidin staining. These effects of HpBE were found to be associated with the modulation in gene expression related to Wnt/β-Catenin and JAK-STAT signalling. Overall, present study underlines the protective potential of HpBE against SDS-induced IBD-like etiologies in *D. melanogaster* model, and lays a foundation for further research to correlate these findings with clinical observations.

## Graphical Abstract

**Figure:**
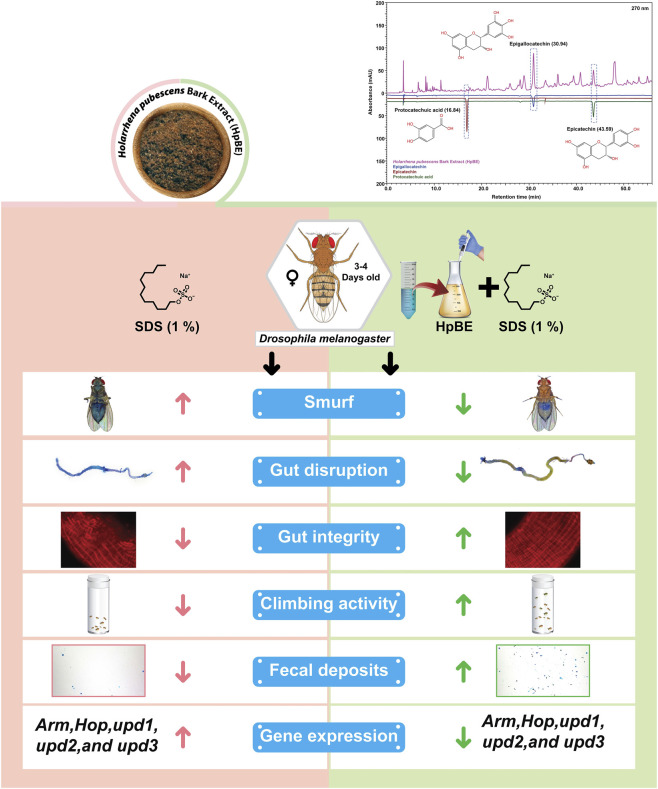

## 1 Introduction

Inflammatory bowel disease (IBD) is a chronic and relapsing inflammatory condition affecting the gastrointestinal tract. It is marked by sustained intestinal inflammation, impairment of epithelial barrier integrity, dysregulated immune responses, and disturbances in the gut microbiota (Saez et al., 2023). People with IBD frequently suffer from symptoms such as abdominal pain, diarrhea, rectal bleeding, weight loss, and fatigue, all of which markedly reduce quality of life (Mitchell et al., 1988; Khan et al., 2024). The incidence of IBD has increased substantially worldwide, with a notable rise in developing nations, positioning it as an emerging public health challenge (Kaplan and Ng, 2017). Despite considerable advances in research, the precise etiologies of IBD remains unclear. Loss of intestinal barrier function permits the translocation of microbial antigens and toxins into underlying tissues, leading to exaggerated immune activation and chronic inflammatory responses (Rogers et al., 2023). Prolonged inflammation results in epithelial injury, heightened oxidative stress, and an elevated risk of complications, including colorectal cancer (Bardelčíková et al., 2023). Although multiple therapeutic strategies such as corticosteroids, immunosuppressive agents, and biologics have been currently employed, their use is often limited by adverse effects, high costs, and inconsistent clinical efficacy (Hafez et al., 2025). Conventional pharmacological management of IBD primarily relies on 5-aminosalicylic acid (5-ASA) and Dexamethasone (Gordon et al., 2011), associated with significant adverse effects like obesity, hyperplasia, infections and adrenal suppression (Poetker and Reh, 2010) particularly with long-term use (Achit et al., 2022). Many patients experience treatment resistance, relapse, or long-term toxicity, highlighting the limitations of existing therapies. An increasing number of studies highlight the protective potential of natural products obtained from diverse plant sources, which possess strong immunomodulatory, anti-inflammatory and antioxidant properties, along with fewer adverse effects compared to conventional treatments (Peng et al., 2023). Notably, many plant-derived compounds exert their beneficial effects by regulating key intracellular signaling, including JAK/STAT and Wnt/β-Catenin pathways. Consequently, there is an urgent need to develop safer, cost-effective, and multi-targeted therapeutic approaches that address the complex mechanisms underlying IBD. Several reports have demonstrated that phytochemicals such as phenolic compounds and flavonoids exhibit potent anti-inflammatory activities by modulating the production of pro-and anti-inflammatory mediators, including IL-1, IL-6, IL-10, TNF-α, NF-κB, nitric oxide (NO), inducible nitric oxide synthase (iNOS), and cyclooxygenase-2 (COX-2) (Olędzka and Czerwińska, 2023). The medicinal use of plants as valuable sources of protective agents for disease treatment and prevention has been recognized for centuries and remains widely practiced across the globe.

One such herb, *Holarrhena pubescens (syn. Holarrhena antidysenterica)*, commonly known as Kutaj or Kurchi, native to arid forest regions worldwide, including India. Kutaj bark extract has a longstanding history of its use in traditional Indian medicine for the management of dysentery, diarrhea, and intestinal parasitic infections (Tiwari et al., 2024). Various parts of the plant, particularly the bark, have been reported to possess antimicrobial, anti-inflammatory, and analgesic properties and are used in the treatment of conditions such as amoebiasis, chronic bronchitis, boils, and ulcers (Bala et al., 2022). The bark of *H. pubescens* contains several bioactive constituents, including gum, resin, tannins, lupeol, digitogenin glycoside holadysone, and multiple alkaloids. Current study utilized *Drosophila melanogaster* as a model organism to examine the effect of *H. pubescens* Bark Extract (HpBE) against sodium dodecyl sulfate (SDS)-induced inflammatory bowel disease (IBD)-like conditions. 1% SDS have previously been reported to cause epithelial damage and intestinal inflammation (Zhang et al., 2020) and are widely used to induce IBD-like conditions across different model organisms (Li et al., 2012).

*Drosophila melanogaster* represents one of the important model organisms and has made fundamental contributions to different areas of biology (Ugur et al., 2016). The digestive system of *Drosophila* contains the foregut, proventriculus, midgut and hindgut, and the intestinal function of *Drosophila* is similar to that of mammals (Xiao et al., 2016; Guo et al., 2022). *Drosophila* midgut harbors multipotent adult stem cells that are essential to renew the gut in homeostatic conditions (Hung et al., 2021; Boumard and Bardin, 2021). Comparative genomic studies estimate that up to 75% of the human genes implicated in diseases are conserved in *Drosophila* (Reiter et al., 2001). In addition, the signaling pathways involved in development of IBD, such as the JAK/STAT, Wnt/β-Catenin and Notch pathways, are highly conserved from *Drosophila* to human beings (Suzuki et al., 2020).

Multiple studies have shown that SDS or DSS-induced intestinal damage is regulated by several metabolic signaling including Wnt/βCatenin and JAK/STAT pathways (Wei et al., 2022). These established pathways formed the rationale for the present study to further evaluate the possible protective effects of HpBE against SDS-induced IBD-like conditions by examining intestinal integrity at both cellular and molecular levels in *Drosophila melanogaster*. Synesthetic steroidal compound, Dexamethasone was used as concurrent positive/method control throughout the study.

## 2 Materials and methods

### 2.1 Chemicals and reagents

HpBE (Batch number: PRF/CHI/0625/0491) was sourced Divya Pharmacy, India. Sodium dodecyl sulphate (Cat # MK1M71170L) was purchased from Emplura, India. Dexamethasone (Cat #D1961) was purchased from TCI, India. Fast green FCF dye (Cat # 42053) was procured from Loba Chemie, India. DAPI (Cat # H-1200) was purchased from Vectashield, India. Trypan blue staining dye (0.4%) (Cat # 15250-061) was purchased from Gibco, India. Epigallocatechin (Potency: 99.66%) was procured from Sigma Aldrich, India, Epicatechin (Potency: 97.0%) was purchased from MCE, India and Protocatechuic acid (Potency: 92.60) was purchased from Natural Remedies, India. Agar-Agar Type I (Cat # GRM666) and methyl-paraben (Cat # GRM 1899) were procured from HiMedia, India. Maize powder and Sulphur less sugar was purchased from Patanjali Foods Ltd., India. Dry yeast powder was purchased from Brew Lab Food and Beverage Pvt. Ltd., India. HPLC grade acetonitrile (Cat # A2104), methanol (Cat #M0275) and acetic acid (Cat # DI40741529) was procured from Rankem, India. Deionized water was obtained from a Milli Q system (Millipore, Billerica, MA, United States).

### 2.2 Phytometabolite profiling of HpBE using UHPLC

Quantitative analysis of marker compounds of HpBE was conducted using Nexera-XR UHPLC system (Shimadzu, Japan) equipped with a quaternary pump (Nexera XR LC-20AD XR), diode array detector (SPD-M20A), autosampler (Nexera XR SIL-20AC XR), degassing unit (DGU-20A5R), and column oven (CTO-10AS VP). VDSpher PUR 120 C18-U column (4.6 × 250 mm, 5 µm) was utilized for chromatographic separation. The mobile phase consisted of solvent A (0.1% acetic acid in water) and solvent B (acetonitrile), delivered at a flow rate of 1.0 mL/min using a gradient elution program. Initially, the mobile phase composition was maintained at 100% solvent A and 0% solvent B for 0–5 min. Thereafter, the proportion of solvent B was gradually increased to 5% at 5 min, 8% at 25 min, 12% at 35 min, 15% at 50 min, 18% at 55 min, 25% at 60 min, and 30% at 65 min. The column temperature was maintained at 30 °C throughout the analysis. Standard and test solutions were injected at a volume of 10 μL, and detection was carried out at 270 nm.

Standard stock solutions of Epigallocatechin (Potency: 99.66%), Epicatechin (Potency: 97.0%) and Protocatechuic acid (Potency: 92.60%) were prepared by dissolving accurately weighed standards in methanol individually (1000 μg/mL). To prepare a 50 ppm standard mix working solution, 0.05 mL of the 1000 μg/mL standard stock solution was diluted in 1 mL methanol.

To prepare hydromethanolic (HM) HpBE powder, 200 g of powdered raw material (*Holarrhena pubescens* bark) was transferred to three-neck round bottom flask for extraction. 1 L of solvent (water: methanol (50: 50) was added to the flask containing material. The extraction was carried out using reflux at 70 °C for 3 h. After that the material was filtered and the remaining material was re-extracted twice under same extraction conditions. Filtrate from all three washes was collected and reduced to dryness using rotary-evaporator (Buchi R-300, Switzerland). The extracted yield was 22.64% w/w. 0.25 g HM HpBE powder was transferred to a 5 mL volumetric flask. Subsequently, a methanol and water mixture in a 50:50 v/v ratio was added to the flask. The resulting solution was allowed to sonicated for 30 min. The solution was then allowed to cool and diluted with same diluent to achieve the desired volume. Prepared solution was then centrifuged for 5 min at 10,000 rpm and filtered using a 0.45 μm nylon filter. The filtered solution was then used for analysis.

### 2.3 Maintenance of *Drosophila melanogaster* culture

The wild-type *Drosophila melanogaster*, strain (Oregon R^+^) was obtained as a generous gift from Dr. Naveen Kumar Gautam, Urology Department, Sanjay Gandhi Postgraduate Institute of Medical Sciences, Lucknow, India. Flies were maintained at 24 °C ± 1 °C under a 12 h light/dark cycle on standard *Drosophila* medium composed of agar-agar type I (1.5 g), maize (17 g), sugar (15 g), yeast (6 g), propionic acid (1 mL), and methyl-paraben (1 g) per 360 mL of food.

### 2.4 HpBE treatment schedule

Female *Drosophila melanogaster* were used in the inflammatory bowel disease (IBD) model because they exhibit relatively stable feeding behavior and reduced variability in food intake compared to males, allowing more consistent assessment of gut injury and treatment responses (Zeng et al., 2011). For treatment, approximately 20 female flies per vial were pre-treated with HpBE at concentrations of 3, 10, and 30 μg/mL in *Drosophila* standard diet for 48 h and then co-treated with HpBE along with 1% SDS for 48 h. SDS (1%) was prepared in 5% sucrose solution. Prior to treatment, female flies were starved for 2 h before transferring them into control, HpBE treatment or Dexamethasone treatments groups. Dexamethasone (30 µM) was used as a positive control drug in all experiments.

### 2.5 HpBE tolerability assessment in *Drosophila*

To assess the tolerability of HpBE, ∼3–4 days old adult female flies (20 flies/vial) were transferred to the *Drosophila* standard food containing different concentrations of HpBE (1, 3, 10, 30, 100 and 300 μg/mL) for 7 days. Number of dead *Drosophila* were counted after 7 days treatment to determine optimal concentration of HpBE to be used for further experimentation.

### 2.6 *Drosophila* survival assay

Survival assay was performed as per previously published protocol (Wei et al., 2022). Briefly, newly emerged ∼3–4 days old female flies were starved for 2 h and transferred into vials containing filter paper soaked with 5% sucrose, HpBE at concentrations 3, 10 and 30 μg/mL or 30 µM Dexamethasone, along with 1% SDS. The control group flies were shifted to vials that held filter paper saturated with 5% sucrose alone. The filter papers with solutions were changed once every 24 h and flies were observed until death in each experimental group.

### 2.7 Measurement of food consumption and weight of *Drosophila melanogaster*

The Capillary feed assay (CAFE) was performed to measure the amount of food consumed by the flies. For this assay, 20 µL microcapillaries with 1 µL mark filled with 5% sucrose solution containing Fast green FCF dye was carefully inserted in the micropipette tip (10 µL volume). The assembled microcapillaries in micropipette tip was then placed in a sponge bung to seal the glass vial (Diegelmann et al., 2017). The capillary ends were positioned inside all vials at the same level to avoid bias that could occur if the food sources were located at different heights (3–4 cm from the lid). The upper end of the colored liquid was marked as the mark^beginning^, and the remaining liquid in the capillary after completion of the experiment was marked as the mark^end^ (Diegelmann et al., 2017). A moist filter paper was placed at the bottom of each vial to maintain humidity. Flies were placed in the vials for 24 h, after which the amount of food consumed was measured between mark^beginning^ and mark^end^ on the capillary using a digital Vernier calliper (Diegelmann et al., 2017). To exclude the effect of evaporation on food intake, calculate mean evaporation in the control vials without flies. Subtract this mean value from the value obtained for food consumption by the flies. Total food consumption per fly was determined as:

Food consumption (µL) = (Food uptake [µL] - Evaporative loss [µL])/total number of flies in the vial (Diegelmann et al., 2017).

Besides, 10 flies per vial were weighed before the treatment, as well as after treatment, and the weight change in each group was calculated using the following formula:

Body weight change = Body weight after treatment/the body weight before treatment (Sun et al., 2025).

### 2.8 Assessment of *Drosophila* faecal deposits

*Drosophila* excretion assay was performed using previously established protocols with some minor modifications (Urquhart-Cronish and Sokolowski, 2014). Briefly, flies from each group (Untreated control, SDS alone, HpBE co-treated and Dexamethasone-co-treated), were starved for half an hour and transferred to the fast green FCF dyed food for 1 h. Thereafter, flies were transferred to the empty Petri plates and remained undisturbed for next 24 h. After 24 h, flies were discarded and the faecal deposits on the lid, bottom and walls of Petri plate were counted under stereomicroscope (Leica Ivesta, Germany).

### 2.9 Determination of *Drosophila* climbing ability

The climbing assay was carried out with slight modifications from previously established protocol (Kauts et al., 2023). 30 female flies were placed into a vertical transparent 25 mL measuring cylinder and gently tapped to bring all the flies at the bottom, after which their upward locomotor activity was monitored for 12 s. Flies that climbed past the 10 cm mark were counted. Climbing ability was expressed as the percentage of flies that crossed the 10 cm line in 12 s. The percentage of flies above and below the mark (n^top^ and n^bot^) were calculated relative to the total number of flies (n^tot^) (Kauts et al., 2023).

### 2.10 Assessment of HpBE consumption in *Drosophila* through LCMS/MS-TQ

To confirm the consumption of HpBE by *Drosophila*, analysis was performed on LC-MS/MS-TQ using lysates of untreated control flies as well as flies fed with 3, 10, 30, 100 and 300 μg/mL of HpBE. Additionally, consumption of HpBE (300 μg/mL) by *Drosophila* was also assessed in the presence of SDS. *Drosophila* lysates were prepared in 700 µL of methanol using ∼300 mg of flies per group. LC-MS/MS analysis was performed using an Agilent Technologies (United States) LC-MS/MS TQ G6475A system equipped with and Agilent Infinity Lab Poroshell 120 EC-C18 column (2.1 × 100 mm, 1.9 µm). Chromatographic separation was achieved using a binary mobile phase consisting 0.1% formic acid in water as mobile phase A and 0.1% formic acid in acetonitrile as mobile phase B. The column temperature was maintained at 25 °C, while the flow rate was kept at 0.3 mL/min throughout the analysis. Fly lysates were centrifuged at 10,000 rpm for 5 min and supernatant was used for analysis. Standards Epicatechin (Potency: 97.0%) and Epigallocatechin (Potency: 98.0%), were dissolved in methanol to prepare 1000 ppm standard stock solution. To prepare 10 μg/mL concentration of each standard individually, 100 µL of standard stock solution (1000 ppm) was diluted to 10 mL with methanol. To prepare 1 μg/mL standard mix solution, 100 µL of 10 μg/mL solutions were mixed and diluted to 1 mL with methanol. Further, to prepare 2 ng/mL, 5 ng/mL, 10 ng/mL, 20 ng/mL, 50 ng/mL, and 100 ng/mL linearity solution were prepared from the 1 μg/mL standard mix working solution. An injection volume of 2.0 µL was injected for both the standard and sample solutions. The chromatographic separation was achieved under gradient elution conditions using solvent B as initially maintained at 2% from 0 to 2 min, gradually increased to 5% at 10 min, further raised to 10% at 12 min, and then to 50% at 15 min. Subsequently, the mobile phase composition was returned to the initial condition of 2% solvent B at 18 min and held constant until 20 min for column re-equilibration.

Mass spectrometric detection was done in positive ionization mode using multiple reactions monitoring (MRM). Epigallocatechin was monitored using the precursor ion at m/z 307.1 with product ions at m/z 139.0 and 163.0 at collision energies of 15 and 23 eV, respectively, with a fragmentor voltage of 140 V and dwell times 20 min. Epicatechin was monitored using the precursor ion at m/z 291.1 with product ions at m/z 123.0 and 139.0 at collision energies of 30 and 15 eV, respectively, using a fragmentor voltage of 100 V with dwell times 20 min. Content of Epicatechin and epigallocatechin were quantified in lysate samples, using calibration curve obtained from the standard solutions injected.

### 2.11 *Drosophila* smurf assay

The smurf assay was performed as previously published protocol with minor modifications (Wei et al., 2022). Briefly, post-treatment, flies from each experimental group were transferred to *Drosophila* standard food containing fast green FCF-dye (2.5% w/v) for 2.5 h. After that, flies were taken out from the vials and observed under microscope to differentiate smurfed (leaky gut) and no smurfed (non-leaky gut) flies. The smurfed flies were identified by the green coloration observed in both the thorax and the abdomen of the fly. The images were acquired under a stereomicroscope (Lieca Ivesta, Germany).

### 2.12 Assessment of gut integrity of *Drosophila*

Post treatment, *Drosophila* gut was dissected out in ice cold 1X PBS, and afterwards gut tissues were fixed in 4% paraformaldehyde (PFA) for 15 min. Tissues were then washed with 0.1% PBST three times (10 min each). Gut tissues were incubated with Phalloidin dye (1: 200) at 4 °C for overnight. Subsequently, gut tissues were washed with 1X PBS and mounted in Vectashield mounting medium. All images were acquired on Mantra fluorescence microscope (APEXVIEW fluorescent microscope (Olympus, Japan) using TEXAS RED filter).

### 2.13 Trypan blue dye exclusion assay in *Drosophila*

To evaluate the *Drosophila* gut cell viability, dye exclusion assay (Trypan blue staining) was performed according to the previously published protocol (Kauts et al., 2024). Following HpBE treatment, dissected midgut of *Drosophila* was incubated in a solution of Trypan blue dye (0.4%) for 15 min and washed with 1X PBS multiple times to remove extra staining. The gut was examined using a stereomicroscope (Lieca Ivesta, Germany), and images were acquired to observe the Trypan blue staining in the gut.

### 2.14 Determination of oxidative stress markers in *Drosophila*

To prepare samples for biochemical analysis, 10 flies per group were collected then homogenised in MilliQ water by a using hand homogeniser to prepare lysate. The lysates were centrifuged (Microcentrifuge, ThermoFisher Scientific) at 10,000 rpm for 15 min to collect supernatant. Enzymatic activity like SOD and GSH levels were analysed and further normalised with protein concentration. The readings were taken on Envision multimode plate reader (Perkin Elmer, United States). The protocol for the estimation of antioxidant parameters was followed as reported previously (Balkrishna et al., 2024).

### 2.15 Quantification of mRNA expression using quantitative real-time PCR (qRT-PCR) in *Drosophila*

The mRNA expression analysis was performed as previously described (Balkrishna et al., 2023). The intensity of fluorescence was captured at each cycle using a real time system machine (CFX OPUS 96 realtime PCR SYSTEM) (Bio-Rad, United States). Primers used for the study are mentioned in Table 1. Relative mRNA expression was calculated using 2^−DDCT^ method. *Gapdh* was used as a housekeeping gene for data normalisation.

**TABLE 1:** Primers for RT-PCR analysis.

S.No.	Primers	Forward (5′-3′)	Reverse (5′-3′)
1	*Armedillo (Arm)*	CTA​GTT​GGG​TTT​CGT​CGC​CA	TTG​AGG​CGC​TTT​ACA​CCA​CA
2	Hopscotch *(hop)*	CTC​CAC​TGC​CAT​GGT​CCA​AT	CGA​TCG​CAT​TCA​CGC​ACA​AT
3	*upd1*	GTC​CGC​GAA​TCA​GTC​TCC​AA	ACA​CAC​TTG​CAG​CAC​AAC​AC
4	*upd2*	GCT​CTG​CGA​GAT​CGA​GTT​GA	GCT​GGC​GTG​TGA​AAG​TTG​AG
5	*upd3*	TGA​ACG​AAA​CGC​ACA​GCA​AG	GTG​ATC​CTG​GCC​TTG​TCC​TC
6	*Gapdh*	CTG​GGC​TAC​ACC​GAT​GAG​GAG	CCA​CGA​GAT​TAG​CTT​GAC​GAA

### 2.16 Statistical analysis

All the experiments were performed in 3 independent biological replicates with each replicate contains 20-30 flies, as per the experiment. The statistical analysis was performed using GraphPad Prism 8 (GraphPad Software, United States). Data were presented as mean ± S.E.M. Significance of the difference between different treatment groups was determined by one-way and two-way ANOVA followed by Dunnett’s *post hoc* analysis.

## 3 Results

### 3.1 Phytometabolite analysis of HpBE

**FIGURE 1:**
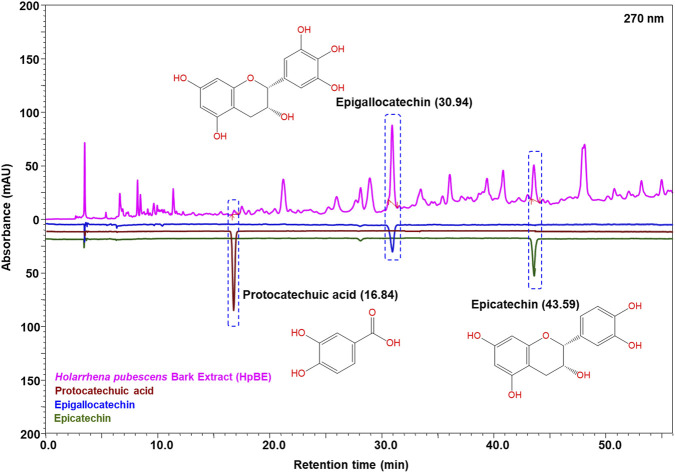
UHPLC profiling reveals the phytometabolites composition of HpBE. UHPLC chromatogram of HpBE. Epigallocatechin standard was represented by blue line, Epicatechin standard was represented by green line, Protocatechuic acid standard was represented by brown line, and HpBE was represented by pink line.

Phytometabolite profiling of HpBE was performed using UHPLC, demonstrated the presence of bioactive compounds such as Protocatechuic acid (RT: 16.70), Epigallocatechin (RT: 30.94) and Epicatechin (RT: 43.59) (Figure 1).

### 3.2 HpBE enhanced survival of SDS-exposed flies

**FIGURE 2:**
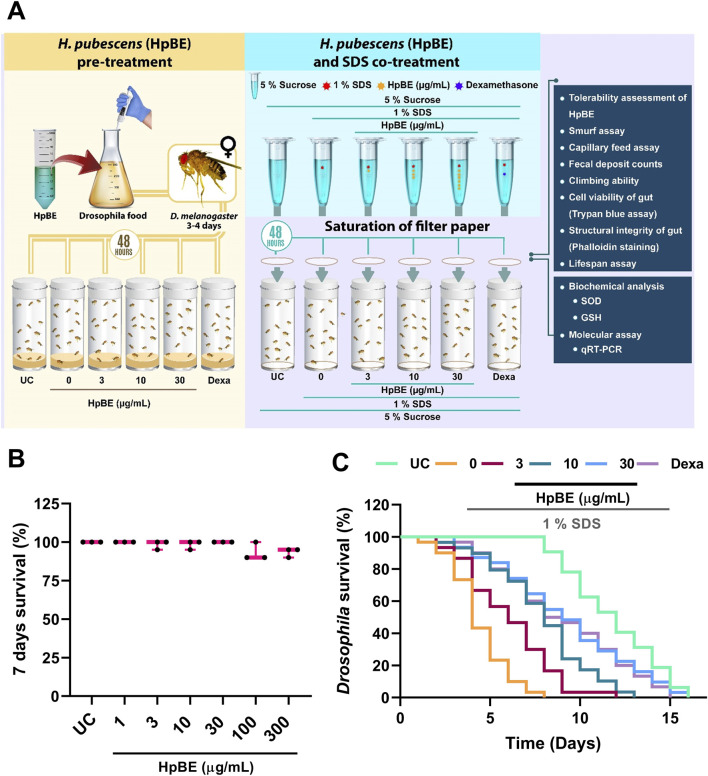
Biocompatible HpBE increased survival of SDS-exposed *Drosophila*. **(A)** Schematic representation of treatment schedule with experimental design to study the effect of HpBE on SDS-induced IBD-like changes in *Drosophila*. **(B)** Shows the effect of 7 days HpBE treatment on *Drosophila* survival (%). **(C)** Kaplan Meier’s curve showing the effect of HpBE (3, 10 and 30 μg/mL) or Dexamethasone (30 µM) on survival of SDS-exposed *Drosophila*. Data was represented as mean ± SEM (n = 3). These experiments were performed in 3 independent biological replicates with each experiment contains 20 flies.

Initially, to assess the tolerability, flies were treated with different concentrations (1, 3, 10, 30, 100, and 300 μg/mL) of HpBE for 7 days (Figure 2A). No significant mortality in flies was observed at any tested conc. of HpBE (Figure 2B). Based on these observations, the concentrations of 3, 10, and 30 μg/mL were chosen for further studies to evaluate the effect of HpBE against SDS-induced IBD-like conditions in *Drosophila*. Further SDS-exposure resulted in significant decrease in lifespan (from 16 days in untreated flies to 8 days in SDS-exposed flies), whereas treatment with HpBE significantly attenuated this SDS-induced decrease in lifespan in conc. dependent manner (Figure 2C). Dexamethasone which was used as a positive control in the study, also showed enhanced lifespan in SDS-exposed flies. These results point towards the protective effects of HpBE against SDS-induced mortality in *Drosophila*.

### 3.3 HpBE increased food consumption, prevented weight loss, improved excretory function and increased climbing ability of SDS-exposed flies

**FIGURE 3:**
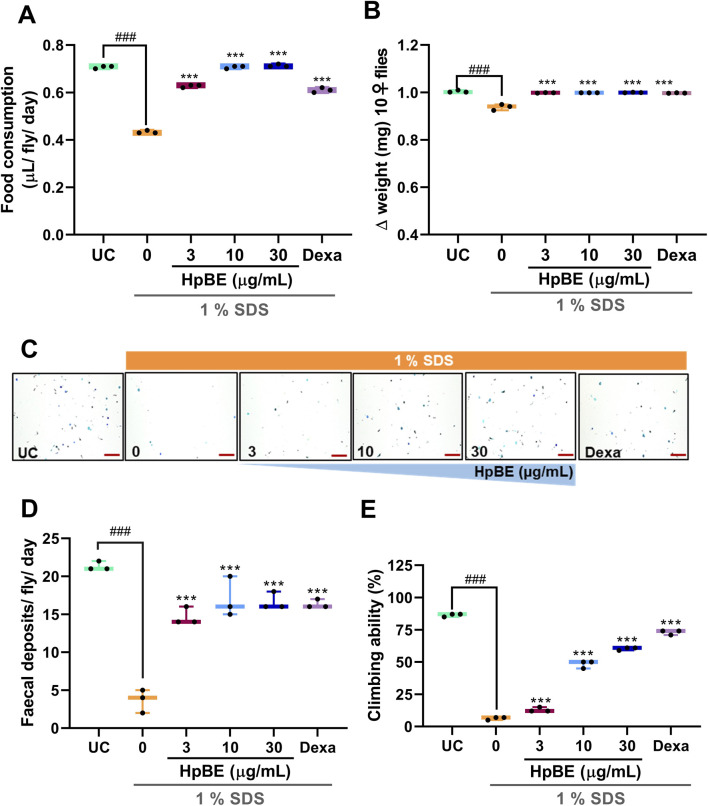
HpBE improved food consumption, body weight, excretion and climbing ability of SDS-exposed flies. **(A)** Shows the effect of HpBE (3, 10, 30 μg/mL) or Dexamethasone treatment on food consumption, **(B)** body weight and **(C,D)** faecal deposits of SDS exposed flies. Scale bar: 300 µm. Data was represented as mean ± SEM. These experiments were performed in 3 independent biological replicates with each replicate contains 20-30 flies, as per the experiment. **(E)** Bar graph representing the effect of HpBE or Dexamethasone on climbing ability of SDS-exposed *Drosophila*. ^###^p < 0.001 represents the statistical significance between untreated group (UC) and SDS-exposed flies whereas ***p < 0.001 represents the statistical significance between SDS-exposed flies and HpBE or Dexamethasone treated flies.

Gut inflammation has been reported to have a direct and measurable impact on food consumption, body weight, and faecal deposition, reflecting impaired digestive and metabolic function (Fändriks, 2017). The present study demonstrated a significant reduction in food consumption and body weight in flies exposed to 1% SDS compared to the untreated control group. In contrast, co-treatment with HpBE significantly increased both food consumption (Figure 3A) and body weight (Figure 3B) at concentrations of 3, 10, and 30 μg/mL. Faecal deposits on Petri plates from SDS-exposed flies were compared with those from untreated control and HpBE co-treated groups, as illustrated in (Figure 3C). Quantitative analysis of the total faecal deposits per fly per day, assessed from the bottom, lid, and cover of the petri plates, revealed that SDS-exposed flies produced fewer faecal deposits than untreated control flies which may result from lower food consumption associated with SDS-induced gut disturbance. Conversely, co-treatment with HpBE along with 1% SDS significantly increased faecal deposition at concentrations of 3, 10, and 30 μg/mL. Moreover, positive control, Dexamethasone also showed more food consumption, body weight and faecal deposits in comparison with 1% SDS-exposed flies (Figure 3D). Climbing ability was used as a functional readout of systemic health following gut inflammation in *Drosophila*. Exposure to 1% SDS markedly reduced climbing performance, with only 7% of flies reaching the 10 cm mark within 12 s, compared to 86% in the untreated control group. Notably, flies treated with HpBE (10 and 30 μg/mL) in the presence of 1% SDS exhibited improved climbing ability, with 48% and 60%, respectively (Figure 3E) Similarly, SDS-exposed flies treated with Dexamethasone also demonstrated a significant improvement in climbing performance.

### 3.4 Determination of HpBE consumption in *Drosophila*

**FIGURE 4:**
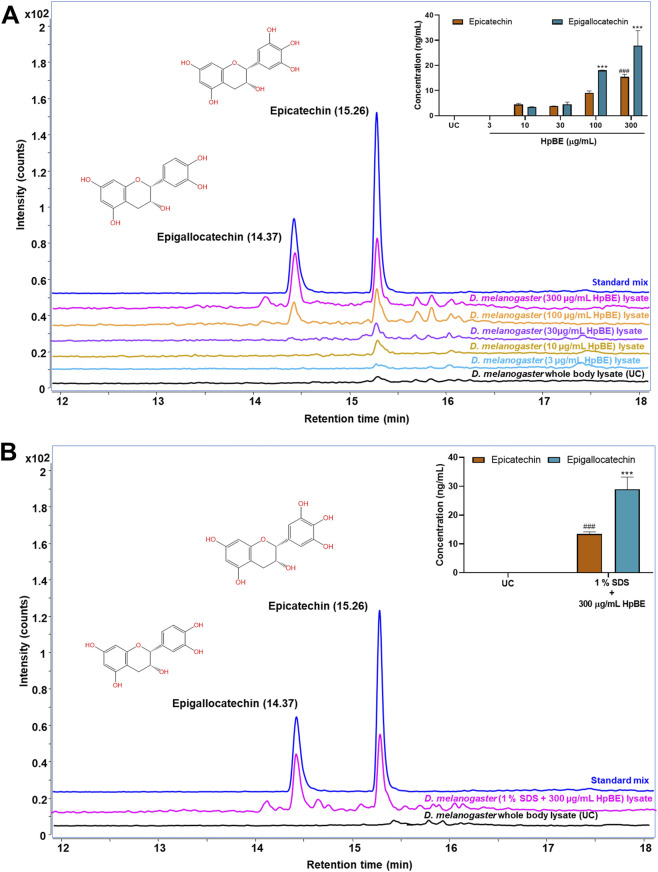
Determination of HpBE consumption in *Drosophila* through LCMS/MS-TQ. **(A)** Overlap chromatogram of standard mix (blue line), 300 μg/mL HpBE *D. melanogaster* lysate (pink line), 100 μg/mL HpBE *D. melanogaster* lysate (yellow line), 30 μg/mL HpBE *D. melanogaster* lysate (purple line), 10 μg/mL HpBE *D. melanogaster* lysate (brown line), 3 μg/mL HpBE *D. melanogaster* lysate (blue line) and untreated control *D. melanogaster* whole body lysate (black line) showing the presence of two phytometabolite compounds: Epicatechin and Epigallocatechin. Bar graph depicts the levels of Epicatechin and Epigallocatechin in the lysates of *Drosophila* fed with different concentration of HpBE (3–300 μg/mL). **(B)** Shows the levels of Epicatechin and Epigallocatechin in the lysates of SDS-exposed *Drosophila* treated with HpBE (300 μg/mL). Data was represented as mean ± SEM (n = 3) and the experiments were performed in 3 independent biological replicates. Significant difference was ascribed as ***p < 0.001 for Epigallocatechin with respect to untreated control (UC) and ^###^p < 0.001 for Epicatechin with respect to untreated control (UC).

To correlate the observed biological activity of HpBE with its consumption by *Drosophila*, LC-MS/MS-TQ analysis was performed on *Drosophila* whole body lysates. Results from this analysis revealed the presence of phytometabolites such as Epigallocatechin (EGC) (RT: 14.37) and Epicatechin (EC) (RT: 15.26) in the flies fed with HpBE at concentrations of 10, 30, 100 and 300 μg/mL (Figure 4A). However, these phytometabolites were not detected at the lowest tested concentration of 3 μg/mL as these levels were below the detection limit of LC-MS/MS-TQ platform, used for analysis. Specifically at 10 and 30 μg/mL HpBE concentrations, these phytometabolite uptake results correlated well with the observed biological effects of HpBE, in SDS-exposed *Drosophila* (Figure 4A). Further, the identification of EGG and EC in the lysates of SDS-exposed *Drosophila* treated with HpBE (300 μg/mL) confirmed that flies were actively consuming HpBE even in the presence of SDS (Figure 4B).

### 3.5 HpBE suppressed smurf phenotype in SDS-exposed *Drosophila*

**FIGURE 5:**
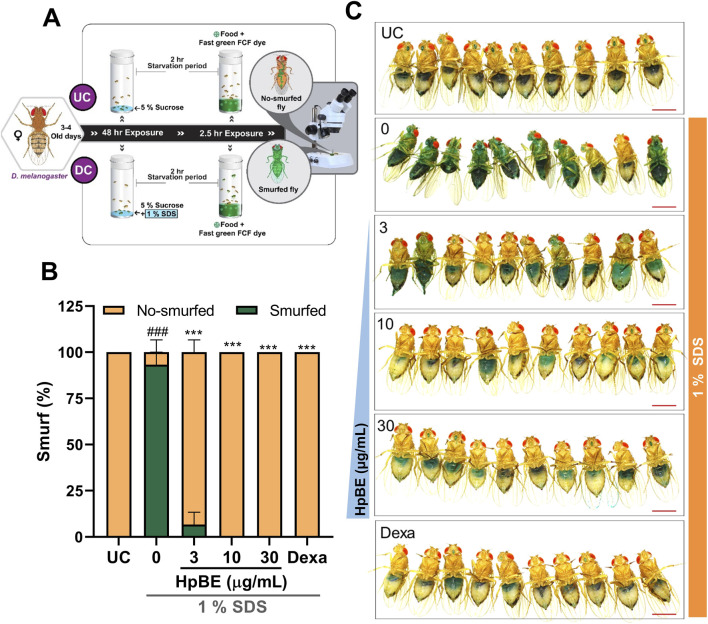
HpBE prevented SDS-induced increase in gut permeability of *Drosophila melanogaster*. **(A)** Schematic representation of experimental paradigm to examine the effect of HpBE on SDS-induced leaky gut phenotype, through smurf assay. **(B)** Shows the smurf percentage of untreated flies (UC), 1% SDS-exposed flies, HpBE (3, 10, 30 μg/mL) treated flies and Dexamethasone (30 µM) treated group. **(C)** Fly images showing gut permeability of untreated flies (UC), 1% SDS-exposed flies, HpBE (3, 10, 30 μg/mL) treated flies and Dexamethasone (30 µM) treated group, representing the leaky and non-leaky gut. Scale bar: 1 mm. Data was represented as mean ± SEM. The experiment was performed in 3 independent biological replicates and each replicate contains 10 flies. The statistical significance represented as ^###^p < 0.001 as compared to untreated control (UC), in contrast ***p < 0.001 represented statistical significance with respect to 1% SDS.

Smurf assay was performed to evaluate if the fly gut remained healthy or became permeable after exposure to SDS. When intestinal barrier integrity is disrupted due to inflammation, aging, infection, toxin exposure, or IBD-like conditions, the orally ingested dye escapes from the gut into the hemocoel, producing a characteristic whole-body blue coloration referred to as the “smurf” phenotype as shown by schematic representation in (Figure 5A) (Rera et al., 2012). In the present study, 93.3% smurfed flies were observed following exposure to 1% SDS, whereas no smurf phenotype was detected in untreated control flies. Treatment with HpBE ameliorated this effect, with only 6.6% of flies showing the smurf phenotype at 3 μg/mL, while no smurfed flies were observed at concentrations of 10 and 30 μg/mL (Figures 5B,C). Similarly, no smurf phenotype was observed in SDS-exposed flies treated with Dexamethasone. These observations suggests that HpBE exhibited the potential to prevent SDS-induced formation of leaky gut, a hallmark of IBD.

### 3.6 HpBE improved gut integrity and reduced oxidative stress in SDS-exposed flies

**FIGURE 6:**
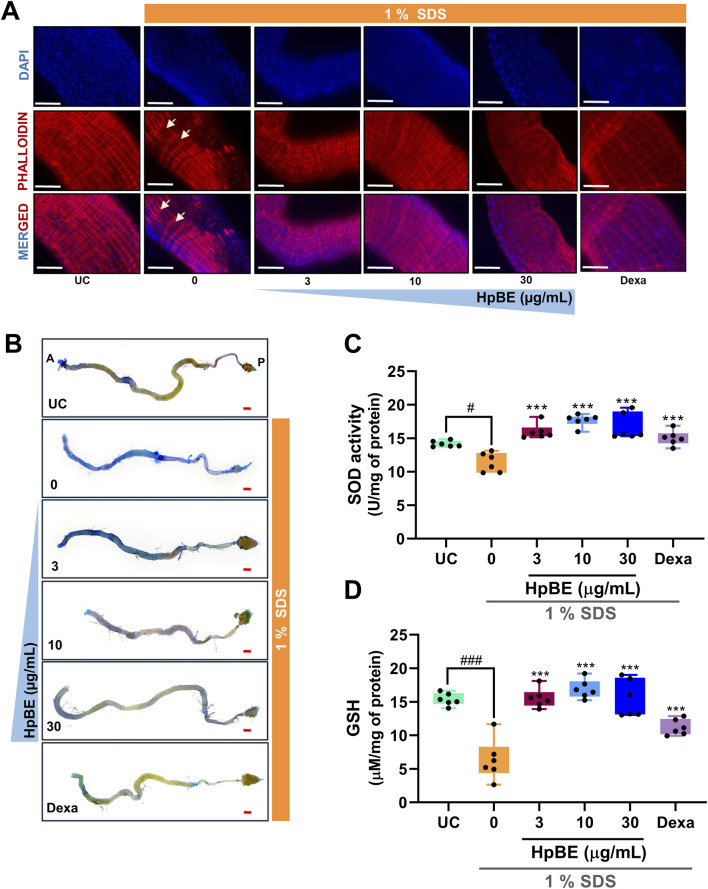
HpBE prevented SDS-induced loss of gut integrity in *Drosophila*. **(A)** Phalloidin staining showing the effect of HpBE (3, 10, 30 μg/mL) or Dexamethasone on SDS-induced alterations in gut cytoskeleton structure of *Drosophila melanogaster* **(B)** Trypan blue dye exclusion assay depicting the effect of HpBE (3, 10, 30 μg/mL) or Dexamethasone on SDS-induced decrease in gut epithelial cell viability in *Drosophila*. Scale bar: 200 µm. **(C,D)** Graphs represent the effect of HpBE (3, 10, 30 μg/mL) or Dexamethasone on SDS-induced changes in SOD activity and GSH levels in *Drosophila*. Data was represented as mean ± SEM (n = 3) and the experiment was performed in 3 independent biological replicates. ^#^p < 0.05 and ^###^p < 0.001 represent the statistical difference between untreated group (UC) and SDS-exposed flies whereas ***p < 0.001 represents the statistical significance between SDS-exposed flies and HpBE or Dexamethasone treated flies.

Loss of gut integrity allows toxins, microbes, and inflammatory mediators to enter the haemolymph, thereby triggering systemic inflammation. Phalloidin staining revealed markedly distorted actin filaments in the midgut of SDS-exposed flies compared to the untreated control group, indicating inflammation-induced cytoskeletal disruption associated with oxidative stress and epithelial cell injury. In contrast, treatment with HpBE restored the intact and organized morphology of actin filaments (Figure 6A). Similarly, Dexamethasone treatment also recovered the disrupted midgut integrity in SDS-exposed flies.

Further, Trypan blue dye exclusion assay demonstrated reduced cell viability in the SDS-exposed midgut, as evident from increased dye permeability throughout the gut. Notably, co-treatment with HpBE at concentrations of 10, and 30 μg/mL restored midgut cell viability (Figure 6B). Additionally, exposure of *D. melanogaster* to 1% SDS resulted in a marked decrease in antioxidant defence mechanism, reflected by decreased superoxide dismutase (SOD) activity and reduced levels of glutathione (GSH). However, co-treatment with HpBE elevated GSH levels and SOD activity at concentrations of 3, 10, and 30 μg/mL (Figures 6C,D). Positive control, Dexamethasone also showed improved gut integrity, and elevated GSH levels and SOD activity in 1% SDS-exposed flies.

### 3.7 HpBE modulated gene expression related to Wnt/catenin and JAK/STAT signalling pathways in *Drosophila*

**FIGURE 7:**
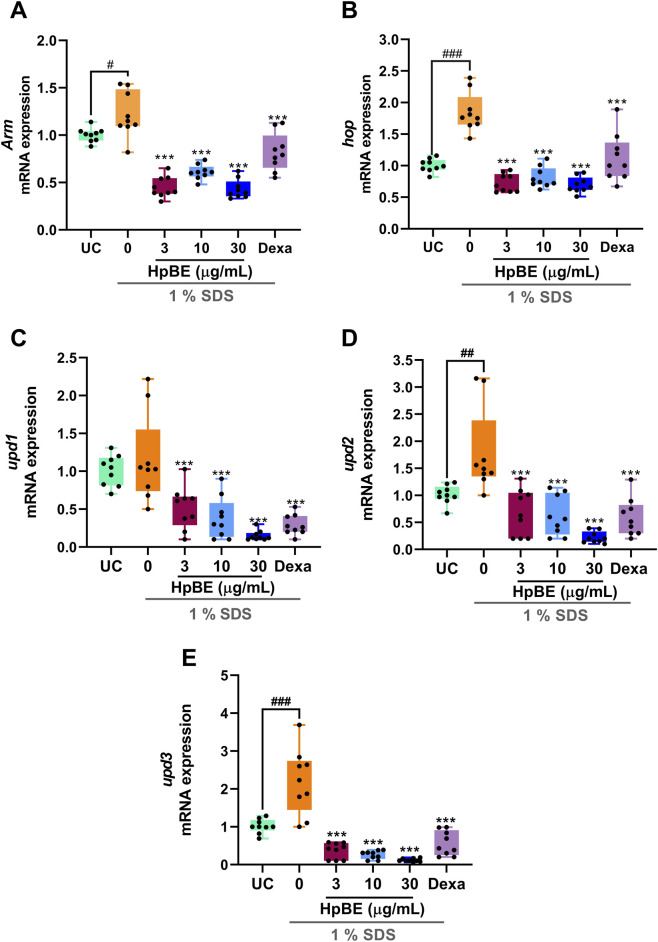
HpBE normalised mRNA expression of genes related to Wnt/β-Catenin and JAK/STAT signalling pathways. Box plots illustrating the effect of HpBE (3, 10, 30 μg/mL) or Dexamethasone on SDS-induced changes in mRNA expression of **(A)** *Arm* **(B)** *Hop* gene **(C)** *upd1* **(D)** *upd2* **(E)** *upd3*. Data was presented as mean ± SEM (n = 3). The experiment was performed in 3 independent biological replicates with each replicate contains 3 technical replicates. ^#^p < 0.05, ^##^p < 0.01, and ^###^p < 0.001 represents the statistical significance between untreated group (UC) and SDS-exposed flies whereas ***p < 0.001 represents the statistical significance between SDS-exposed flies and HpBE or Dexamethasone treated flies.

The activation of Wnt/β-catenin signalling pathway is crucial for development of chronic IBD. This signalling is vital for differentiation of intestinal stem cells. Armadillo (*Arm*) functions as the homologue of vertebrate β-catenin in *Drosophila melanogaster* (Yan et al., 2023). Therefore, the effect of HpBE on SDS-exposed changes in mRNA expression of *Arm* was evaluated. SDS-exposure led to a significant increase in the mRNA expression of *Arm*, indicating its potential to modulate Wnt/β-catenin signalling. This increase in mRNA expression of *Arm* was significantly suppressed in SDS-exposed flies treated with HpBE (Figure 7A). Apart from Wnt/β-catenin signalling, activation of JAK/STAT signalling has also been shown to be associated with the pathogenesis of IBD (Yan et al., 2023). The hopscotch (*hop*) gene found in *Drosophila* encodes Janus Kinase (JAK). SDS-exposed flies exhibited increased mRNA expression of *hop*. However, co-treatment with HpBE significantly blunted this SDS-induced increase in mRNA expression of *hop* (Figure 7B). Additionally, the effect of HpBE on mRNA expression of ligands (*upd1, upd2 and upd3*) known to activate JAK/STAT signalling was also assessed in SDS- exposed flies. SDS exposure led to significant increase in the mRNA expression of *upd2* and *upd3*. However, HpBE treatment significantly prevented this increase in the mRNA expression of *upd2* and *upd3*. SDS exposure to *Drosophila* also enhanced the mRNA expression of *upd1* gene (although not statistically significant), which was prevented by HpBE (Figures 7C–E). These results suggest that HpBE-mediated protection against acute SDS-induced damage in the *Drosophila* is associated with modulation of gene expression related to Wnt/Catenin and JAK/STAT signalling pathway.

## 4 Discussion

IBD is a chronic inflammatory disorder of gastrointestinal tract, classified into Crohn’s disease and ulcerative colitis (Torres et al., 2017). The exact etiology of IBD is multifactorial involving environmental factors, immune dysfunction, genetic susceptibility and gut microbiota dysbiosis leading to dysregulated mucosal immune response. Current treatment options for IBD mainly aim to achieve symptom remission and preventing long-term complications such as hospitalization, surgery and colorectal cancer (Jeong et al., 2019; Chapman et al., 2020). While the use of conventional therapies such as 5-aminosalicylates, corticosteroids, and immunomodulators are essential for some patients, their application is frequently limited by adverse effects and inconsistent efficacy, emphasizing an urgent need for effective and safer options. Present study explored the potential of medicinal plant *Holarrhena pubescens* Bark Extract (HpBE) against SDS-induced IBD-like etiologies using *Drosophila* model.

The single-layered simple epithelium of the gastro-intestinal tract controls nutrient uptake, coordinates metabolism and protects from luminal components including chemical poison or pathogens (Ley et al., 2006). Accordingly, the maintenance of intestinal barrier function, including its metabolic and immune functions, is essential for organismal health. Epithelial homeostasis is dependent on a balance of intestinal stem cell (ISC) self-renewal, progenitor differentiation, cell shedding and apoptosis (Radtke and Clevers, 2005). Phytometabolite characterization of HpBE through UHPLC revealed the presence of key phytometabolites, namely, Protocatechuic acid, Epigallocatechin (EGC) and Epicatechin (EC). Catechin compounds have shown to possess anti-inflammatory, anti-tumor, anti-oxidation and anti-bacterial properties (Wang et al., 2025). Therefore, presence of EGC and EC in HpBE could contribute to these protective effects offered by it.

Sodium dodecyl sulphate (SDS) has been identified as a chemical inducer for modelling IBD-like pathologies in *Drosophila melanogaster*. SDS-induced IBD-like phenotype in *Drosophila* is characterised by severe intestinal inflammation, barrier disruption, and high mortality. The results from present study demonstrated that HpBE was not only tolerated well by *Drosophila*, it also prevented SDS-induced reduction in the lifespan. The flies that were fed with different concentrations of HpBE, demonstrated the presence of EGC and EC that were identified in HpBE itself, confirming the successful uptake of HpBE by *Drosophila*. However, at the lowest tested concentration of 3 μg/mL, the HpBE phytometabolites were not detected, largely due to being below the detection limit of LC-MS/MS-TQ platform. Therefore, the phytometabolite uptake data may not be interpreted that these marker compounds play a role in the protective effects noted at lower tested concentration, such as 3 μg/mL of HpBE. However, at 10 and 30 μg/mL concentrations, the phytometabolite uptake results correlated well with the observed biological effects of HpBE, particularly, in SDS-exposed *Drosophila*. Exposure to SDS has been reported to induce intestinal injury in *Drosophila melanogaster*, characterized by the appearance of the smurf phenotype and increased oxidative stress (He et al., 2022; Wei et al., 2022). Higher incidence of the smurf phenotype reflects compromised gut barrier function and increased intestinal permeability, indicating the presence of gut inflammation and oxidative damage (Salazar et al., 2023). Breakdown of the intestinal barrier permits the translocation of microbial components, toxins, and inflammatory molecules into the haemolymph, thereby triggering systemic inflammatory responses (Khaerani et al., 2024). The results from this study demonstrated that HpBE could prevent the loss of gut integrity caused by SDS, as indicated by reduced number of smurfs. This protective effect of HpBE could be attributed due to the presence of Epicatechin which has been shown to possess strong anti-inflammatory activity (Zhang et al., 2016). Further, the gut protective effect of HpBE could also be due to the presence of phenolic phytometabolite, Protocatechuic acid as it has previously been reported to prevent DSS-induced disruption of intestinal epithelial barrier through redistribution of tight junction proteins in C57BL/6 mice.

In addition, Epigallocatechin which is also present in HpBE has been reported to mitigate disease manifestations associated with LPS-induced sepsis and systemic inflammation by modulating key inflammatory and antioxidant pathways. Its protective effects involve the regulation of NF-κB and Nrf2/ARE-mediated transcription, along with inhibition of MAPK and PI3K/Akt signalling cascades (Al-Sayed and Abdel-Daim, 2018). Gut inflammation in *Drosophila* has been strongly associated with decreased locomotory activity (Keshav et al., 2022). Climbing activity in flies is an innate response and is a strong indicator of healthy flies (Linderman et al., 2012; Keshav et al., 2022). A drastic reduction in climbing activity of SDS-exposed flies was observed which was significantly restored by HpBE underlining its potential in preventing sickness related to gut inflammation. Further, the disruption of intestinal integrity in *Drosophila* has been shown to be associated with reduced feed uptake, excretory dysfunction and weight loss (Yan et al., 2023). Consistently, findings from this study demonstrated decreased food consumption, weight loss and excretion (decreased faecal output) in flies exposed to SDS. However, flies treated with HpBE normalised feeding behaviour, protected excretory function and prevented SDS-mediated weight loss, confirming its role in maintaining gut integrity.

The cytoskeletal network in the gut has been reported to be involved in the regulation of epithelial barrier function (Banan et al., 2000; Perrin and Matic Vignjevic, 2023). An interesting study by Wan et al., reported the potential of EC and EGC in maintaining gut cytoskeletal integrity by reducing oxidative stress and inflammation that disrupted actin dynamics (Wan et al., 2022). They observed that these polyphenols were found to stabilize F-actin architecture and maintain epithelial cell-cell junctions. By protecting intestinal epithelial cells from stress-induced cytoskeletal disruption, they contribute to the preservation of gut barrier integrity and support normal intestinal functions (Huo et al., 2022). Restored gut integrity and normalised actin filament morphology, along with enhanced anti-oxidant activity in SDS-exposed flies treated with HpBE further strengthens its role in preventing the IBD like etiologies.

The activation of Wnt/β-catenin signalling pathway plays an important role in the development of chronic IBD. This signalling plays a crucial role in regulating the differentiation of intestinal stem cells. Armadillo (*Arm*) serves as a *Drosophila melanogaster* homolog of vertebrate β-catenin. Within the gut, *Arm* plays a dual and conserved role, functions both as structural component of adherens junctions and transducer of the Wingless (Wg/Wnt) signalling pathway (Yan et al., 2023). Through this study, it was demonstrated that, HpBE-mediated intestinal homeostasis in inflammatory environment was associated with modulation of gene expression related to Wnt/β-catenin signalling pathway. The hopscotch (*hop*) gene in *Drosophila* encodes for Janus Kinase (JAK) which is essential for embryonic patterning, segment formation, and larval development. The IBD-like etiologies in *Drosophila* have been found to be associated with activation of JAK/STAT signalling pathway. Yang et al., reported hyperactivation of JAK/STAT signalling pathway in *Drosophila* gut exposed to SDS for 16 h (Yan et al., 2023). In tune with these results, normalised mRNA levels of *hop* by HpBE in SDS-exposed flies highlights its potential to modulate the gene expression related to JAK/STAT signalling. JAK/STAT signalling in *Drosophila* has been shown to be activated by extracellular ligands such as *upd1, upd2 and upd3* (Jiang et al., 2009). The receptor binding of *upd1, upd2 and upd3* to the domeless receptor further leads to the activation of Hopscotch (JAK) kinase which then phosphorylates the cytoplasmic tail of the domeless receptor (Jiang et al., 2009). The phosphorylated receptor acts as a docking site for STAT92E. Once STAT92E binds to phosphorylated receptor, it is phosphorylated by Hopscotch. The phosphorylated STAT92E forms dimer, which translocate into the nucleus and drive the expression of various target genes related to immune response (Jiang et al., 2009). Present study also demonstrated that SDS exposure of 48 h led to a significant upregulation of *upd3* gene expression, thereby validating the inflammatory nature of the model used in the present study. Apart from upd3, assessment of the mRNA expression of other extracellular ligands (*upd1* and *upd2*) of *Drosophila* domeless receptor revealed that HpBE treatment not only prevented SDS-induced increase in the mRNA expression of *upd3* but also decreased the gene expression of *upd1* and *upd2*, suggesting its protective effects towards acute SDS-induced gut inflammation.

## 5 Conclusion

Collectively, present study evaluated the protective potential of HpBE against SDS-induced IBD like etiologies using *Drosophila melanogaster* as a model organism. The finding from present study clearly demonstrated that HpBE increased the survival rate of flies exposed to SDS, protected intestinal integrity, diminished cell death of intestinal epithelial cells and alleviated sickness associated with inflammatory gut. These protective effect of HpBE against SDS-induced intestinal injury were found to be associated with modulation of gene expression related to Wnt/β-Catenin and JAK/STAT signalling. Overall, these findings highlight the protective potential of HpBE against intestinal damage that is common in IBD or other intestinal aliments. Despite these encouraging outcomes, the study has certain limitations. *Drosophila melanogaster* may not fully recapitulate the complexity of mammalian intestinal inflammation. Furthermore, the findings of present study support protection against acute chemically induced injury, but not reversal of established disease. Therefore, additional studies are needed to better understand the effect of HpBE in higher vertebrate models of IBD, including non-clinical toxicity assessment under GLP compliance.

## Data Availability

The original contributions presented in the study are included in the article/supplementary material, further inquiries can be directed to the corresponding author.
